# Systematic Comparison of Different Compartmental Models for Predicting COVID-19 Progression

**DOI:** 10.3390/epidemiologia6030033

**Published:** 2025-07-08

**Authors:** Marwan Shams Eddin, Hussein El Hajj, Ramez Zayyat, Gayeon Lee

**Affiliations:** 1 Department of Systems Engineering and Operations Research, George Mason University, Fairfax, VA 22030, USA; 2 Department of Information Systems and Analytics, Santa Clara University, Santa Clara, CA 95053, USA; helhajj@scu.edu (H.E.H.); rz29@aub.edu.lb (R.Z.); glee2@scu.edu (G.L.)

**Keywords:** COVID-19 pandemic, compartmental models, epidemic forecasting, prediction accuracy, disease progression, healthcare preparedness

## Abstract

**Background/Objectives**: The COVID-19 pandemic highlighted the critical need for accurate predictive models to guide public health interventions and optimize healthcare resource allocation. This study evaluates how the complexity of compartmental infectious disease models influences their forecasting accuracy and utility for pandemic resource planning. **Methods**: We analyzed a range of compartmental models, including simple susceptible-infected-recovered (SIR) models and more complex frameworks incorporating asymptomatic carriers and deaths. These models were calibrated and tested using real-world COVID-19 data from the United States to assess their performance in predicting symptomatic and asymptomatic infection counts, peak infection timing, and resource demands. Both adaptive models (updating parameters with real-time data) and non-adaptive models were evaluated. **Results**: Numerical results show that while more complex models capture detailed disease dynamics, simpler models often yield better forecast accuracy, especially during early pandemic stages or when predicting peak infection periods. Adaptive models provided the most accurate short-term forecasts but required substantial computational resources, making them less practical for long-term planning. Non-adaptive models produced stable long-term forecasts useful for strategic resource allocation, such as hospital bed and ICU planning. **Conclusions**: Model selection should align with the pandemic stage and decision-making horizon. Simpler models are effective for rapid early-stage interventions, adaptive models excel in short-term operational forecasting, and non-adaptive models remain valuable for long-term resource planning. These findings can inform policymakers on selecting appropriate modeling approaches to improve pandemic response effectiveness.

## 1. Introduction

The COVID-19 pandemic, caused by the SARS-CoV-2 virus, has posed numerous challenges to global healthcare systems (e.g., infrastructure, supply chains, human resources, government preparedness, and public health systems) [1]. The unprecedented infection rate overwhelmed healthcare capacities worldwide. Healthcare systems were particularly strained during the initial wave of the pandemic, resulting in shortages of ventilators, personal protective equipment (PPE), and adequately equipped intensive care unit (ICU) beds [2]. This healthcare crisis prompted policymakers and governmental agencies to develop strategies to increase the supply of critical resources, such as ICUs, hospital beds, and ventilators, while establishing interventions to help curb the spread of the virus.

Governments worldwide implemented a variety of short-term policies, such as lockdowns, social distancing, school closures, and mass testing, in addition to promoting hygiene measures and the use of face coverings [3]. In the long term, vaccination campaigns were employed, particularly following the development and approval of COVID-19 vaccines for the different variants. However, the scarcity of resources, especially in the early stages of the pandemic, made the effective implementation of these policies challenging. Strategic decisions on how to allocate these limited resources were essential for managing the pandemic effectively. For example, forecasting models can inform decisions about when to trigger or lift lockdowns, how to distribute vaccines or antiviral treatments across regions, and how to allocate limited hospital resources such as ICU beds and ventilators. This, in turn, necessitated the development of approaches that could accurately estimate the disease’s progression within a population. Accurate predictions of disease progression are critical for mitigating the pandemic and ensuring preparedness, allowing for targeted resource allocation to minimize the damage caused by an emerging pandemic.

Mathematical and forecasting models have played a vital role in understanding and predicting disease transmission within populations. Classical compartmental models, such as SIR (Susceptible, Infectious, Recovered) [4,5] and SEIR (Susceptible, Exposed, Infectious, Recovered) [6,7], have been widely adopted to estimate epidemic dynamics. To better capture the complexities of diseases like COVID-19, several extensions have been proposed, including SEIRD (Susceptible, Exposed, Infectious, Recovered, Dead) [8], SEAIRD (Susceptible, Exposed, Asymptomatic, Infectious, Recovered, Dead) [9], and SIDARTHE (Susceptible, Infected, Diagnosed, Ailing, Recognized, Threatened, Healed, Extinct) [10]. With the purpose of improving the forecasting accuracy, these models categorize the population into mutually exclusive compartments, which interact based on rates such as transmission, recovery, and mortality. Given the limited resources available during pandemics, accurate predictive models not only aid in resource allocation but also play a vital role in informing public health interventions. By controlling disease spread and reducing mortality rates, these models support real-time decision-making, ensuring that resources such as ventilators, ICUs, and PPE are allocated efficiently to prevent overwhelming healthcare infrastructures. The goal of this work is to evaluate the predictive accuracy of several such models and explore how model complexity influences their ability to capture the actual number of infections. This evaluation provides insights for both short-term crisis management and long-term pandemic planning.

Accurate disease predictions are closely linked to the effectiveness of implemented policies. For example, if predictions suggest a low number of infections but actual cases are high, resources will be insufficient, potentially leading to increased mortality rates. Conversely, if predictions suggest high infection numbers, but the actual cases are low, limited resources might be misallocated, leading to surpluses in some areas and shortages in others. Forecasting models can also help hospitals anticipate surges in ICU admissions, allowing staff and resources to be reorganized and essential medications and equipment to be procured in advance, thus reducing the strain on healthcare systems [11].

The main contributions of this paper are as follows:

- We examine the predictive accuracy of a range of models, from simpler models like SIR and SEIR to more complex models such as SIDARTHE and adaptive/non-adaptive SEAIRD.
- We conduct numerical analyses by applying these models to actual COVID-19 data from the U.S.
- We provide insights into the conditions under which each model performs best, offering critical guidance for healthcare policymakers in terms of resource allocation and predicting both short- and long-term pandemic impacts.

The rest of the paper is organized as follows: In Section 2, we present our systematic approach to selecting relevant papers and provide a comprehensive literature review of each considered model. In Section 3, we outline the mathematical framework of each model and conduct a numerical analysis evaluating the predictive accuracy of the models using COVID-19 data from the U.S. Finally, in Section 5, we present our conclusions, discuss the limitations of this research, and suggest directions for future work.

## 2. Materials and Methods

In this section, we begin by outlining our systematic approach for selecting the most relevant studies focused on disease progression prediction. We then narrow our focus to widely used compartmental models (which we will focus on in this paper), providing explanations and situating them within the context of the most pertinent literature.

### 2.1. Literature Search Strategy

Four major databases, namely ScienceDirect, Web of Science, PubMed, and Scopus, were selected for their comprehensive coverage of peer-reviewed literature, particularly in the fields of epidemiology, infectious diseases, and healthcare [12]. To capture a broad range of studies focused on modeling and forecasting approaches related to the COVID-19 pandemic, a combination of relevant keywords was used, including COVID-19, Forecasting, Prediction, Epidemic Model, SIR, SEIR, and SEIRD. This search strategy retrieved over 14,000 research documents addressing COVID-19 management strategies and their application within epidemic modeling frameworks.

**Figure 1:**
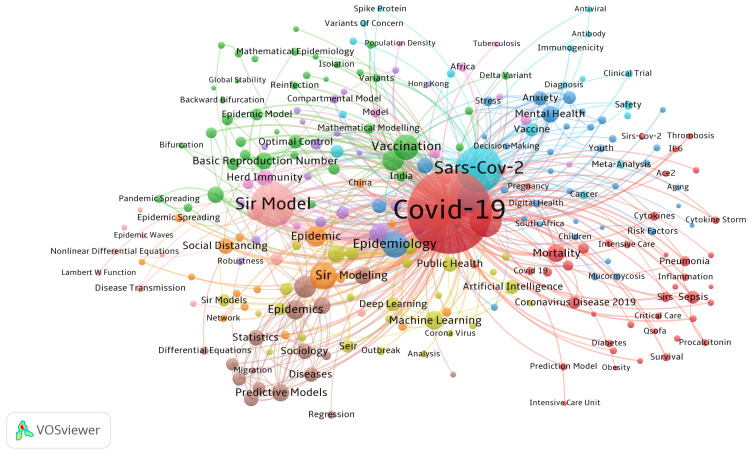
Keyword analysis with VOSviewer.

To focus on the most recent and relevant studies, the literature search was restricted to publications from 2020 onward, aligning with the onset of the COVID-19 pandemic. Furthermore, we limited our search to peer-reviewed journal articles to ensure the quality and reliability of the covered studies. VOSviewer version 1.6.20 [13] was employed to conduct a bibliometric analysis of the selected literature. The tool was used to create a co-occurrence map of keywords, allowing us to visualize the relationships between key terms (see Figure 1). This analysis enabled us to visualize the relationships between key terms and identify major research clusters, offering a clearer understanding of the trends and gaps in the existing literature. The VOSviewer keyword map highlights the modeling and forecasting aspects of COVID-19. Central to the map is the COVID-19 keyword, surrounded by closely connected terms like SIR Model, Epidemic Modeling, and Machine Learning, underscoring the critical role of mathematical and statistical frameworks in predicting the pandemic’s trajectory. To enhance the selection process, specific inclusion and exclusion criteria were established to identify research that contributed directly to predicting disease progression. In this paper, we mainly focus on compartmental models, as they have been widely employed to estimate the evolution of pandemics with reasonable accuracy. As such, the inclusion criteria focused on studies published in peer-reviewed journals that examined COVID-19 epidemic models like SIR, SEIR, SEIRD, and their variants. Preprints were excluded from this review, despite their influence during the early stages of the pandemic, to ensure that only peer-reviewed, validated studies were analyzed. Non-peer-reviewed articles, editorials, commentaries, opinion pieces, book chapters, unpublished work, and research focused purely on theoretical models without real-world healthcare applications were also excluded. For a comprehensive review of COVID-19 progression, mitigation strategies, testing, vaccination, and non-pharmaceutical interventions, see [14,15].

### 2.2. Compartmental Models and Relevant Literature

We begin by defining compartmental models as tools that predict disease progression. Such models divide the population into distinct groups based on infection status, and as such, given initial conditions and calibrated parameters, these models simulate transitions between compartments over time to generate the predicted disease progression.

The SIR model, introduced by [5], divides the population into susceptible (S), infected (I), and removed (R) groups. Despite its simplicity, it offers useful analytical insights [16,17]. Studies show that its predictive accuracy improves with proper parameter tuning [18,19]. Enhancements like Particle Swarm Optimization [20] and time-dependent parameters [21] have further reduced estimation errors. Still, the model’s three-compartment structure often fails to capture complex epidemic dynamics.

The SEIR model extends the classical SIR framework by adding an Exposed (E) compartment, representing individuals who have been infected but are not yet infectious [22]. This captures the latent period between exposure and symptom onset, making the model more realistic. Individuals in the exposed group eventually become infectious or recover. Many diseases modeled by SIR also fit well within the SEIR framework [23]. Thanks to its simplicity and improved predictive power, the SEIR model has been widely applied to forecast the spread of COVID-19 [24,25,26,27,28].

A key limitation of the SIR and SEIR models is the assumption that recovered individuals gain permanent immunity. The SEIRD model overcomes this by adding a Dead (D) compartment [17]. This allows differentiation between those who may lose immunity and become susceptible again and those who have died. For example, COVID-19 immunity has been observed to wane after two to three months [29]. The SEIRD model has been successfully applied to monitor COVID-19 progression, such as in Italy [30].

While SEIR and SEIRD models improve on the basic SIR structure, they generally assume all infected individuals develop symptoms. However, about 15.5% of COVID-19 cases are asymptomatic [31], and these individuals can still spread the virus, complicating prediction and control efforts. Additionally, these models often assign equal interaction rates to symptomatic and asymptomatic individuals, despite symptomatic cases being more likely to self-isolate and reduce contacts.

To address this limitation, ref. [32] developed a model separating symptomatic and asymptomatic compartments, applied to COVID-19 in Heilongjiang, China. Ref. [33] showed that combining testing with contact tracing improves early detection and containment. In the U.S., ref. [34] demonstrated that integrating digital contact tracing with mass testing speeds up identification and isolation, including the identification and isolation of asymptomatic individuals. These methods often assume perfect testing and unlimited resources. To improve this, ref. [35] introduced an SIQRD model with a quarantine (Q) compartment for false positives, separate from true positives in the recovered (R) group. The model allows false positives to return to susceptibility after quarantine and captures multiple COVID-19 waves in the U.S.

The SIDARTHE model by [10] introduces additional compartments that capture different stages of testing and disease severity, including Diagnosed (D), Ailing (A), Recognized (R), Threatened (T), Healed (H), and Extinct (E). In this framework, symptomatic and asymptomatic individuals are represented by the I and A compartments, respectively to maintain consistency with our notation, we reversed the roles of *I* and *A* from the original model [10]. This level of detail allowed the model to significantly improve the accuracy of COVID-19 progression forecasts in Italy.

The compartmental models discussed above are widely used in the literature. However, to the best of our knowledge, a systematic, accuracy-focused evaluation of these models has not been conducted. This work addresses that gap by identifying the conditions under which each model performs best.

To enhance readability and facilitate comparison, Table 1 summarizes the compartmental models discussed in the literature, highlighting their structures, key features, application examples, and limitations.

## 3. Case Study

This numerical study evaluates the predictive accuracy of four compartmental epidemic models, SIR [4], SEIRD [8], SEAIRD [9], and SIDARTHE [10], as introduced in Section 2.2. Using daily COVID-19 data, the models are assessed based on their ability to forecast pandemic trends, which is essential for timely and effective resource allocation. Resources are grouped into short-term, such as masks and testing kits, and long-term, such as ventilators and hospital beds, based on the response strategies they support. The study investigates how accurately each model predicts disease progression and whether increased complexity improves performance. It also considers the balance between model detail and practical use, especially when rapid decisions must be made with limited data.

### 3.1. Simplified Mathematical Formulation of Epidemic Models

This section presents the mathematical formulation of each selected epidemic model. This provides a basis for comparing their predictive capabilities and highlights the level of detail each model captures. While we aim to use unified notation, some model-specific terms remain due to varying complexities.

Due to data limitations, we simplified the models presented in the original studies by focusing on disease transmission in the primary compartments discussed in Section 2.2. For example, in the SEIRD model, the original formulation distinguished between hospitalized and non-hospitalized populations, but we used a simplified version that models the entire population without this subdivision, as hospitalization data were not consistently available. Similarly, in the SIDARTHE model, we removed compartments related to testing and lockdown interventions, such as diagnosed or recognized individuals, because we did not have reliable data on testing rates or detection status in the population. We refer readers to Table 2 for a summary of the simplified models. These modifications allow the models to focus on the core transmission dynamics while remaining feasible given the available data. Table 3 summarizes the compartments included in each model to enhance transparency regarding these simplifications.

By simplifying the models, we focus on the main transitions between compartments such as susceptible, exposed, infected, and recovered individuals, while excluding more detailed categories like hospitalization or intervention-related statuses. This choice is guided by the availability and reliability of the data, as well as the objective of capturing the core dynamics of disease transmission. Our goal is to represent the most influential factors, such as infection and recovery rates, while avoiding added complexity from compartments that require extensive or unavailable data. Although this simplification may omit certain aspects, such as healthcare system capacity or the direct impact of public health interventions, it allows the models to remain practical and retain their ability to predict key outcomes like infection peaks and recovery patterns. This simplification means that, given the values of the parameters and initial conditions for each compartment, one can easily run the dynamics via forward substitution (a step-by-step calculation of future states based on current values) to generate the values of each state over time.

### 3.2. Model Calibration and Data Sources

To evaluate the predictive accuracy of the selected compartmental models, we used daily COVID-19 case data from the Johns Hopkins Center for Systems Science and Engineering (CSSE) [36]. The dataset covers the period from 22 January 2020 to 9 March 2023 and includes daily counts of confirmed cases, deaths, and recoveries across the United States, aggregated from state and county-level reports. Due to the incomplete reporting of recoveries, we make the simplifying assumption that all infected individuals either recover or die within 14 days of infection. This assumption aligns with public health isolation guidelines and virological studies indicating that most individuals are no longer infectious after this period. While viral RNA may persist beyond 14 days, live virus is rarely detectable after 8–10 days in mild to moderate cases, with fewer than 5% of individuals remaining infectious by day 15 [37,38]. Moreover, clinical reports suggest that symptoms in non-severe cases substantially subside within two weeks. We acknowledge that this simplification does not reflect prolonged clinical courses observed in severe or critical cases, which are not explicitly modeled in our framework. From a transmission modeling perspective, individuals are treated as epidemiologically and functionally removed after 14 days, consistent with standard interpretations of the “Recovered” compartment. Additionally, we model only symptomatic infections, assuming that reported cases represent symptomatic individuals, with 15.6% of all infections being asymptomatic [31]. The exposed population is estimated to be five times the number of confirmed cases [31,39].

To capture the multiple-wave phenomenon exhibited during the COVID-19 pandemic, driven by viral variants and changing public health policies, we divided the dataset into five distinct 120-day intervals. We chose this 120-day prediction horizon to strike a balance between short-term forecasting and medium-term policy planning. This time frame aligns with typical public health decision-making cycles, such as quarterly planning for intervention strategies, vaccination logistics, and hospital surge preparedness. It is long enough to capture meaningful variation in epidemic dynamics while avoiding the excessive uncertainty associated with long-range forecasts. Our goal is to evaluate model robustness under conditions that are both epidemiologically informative and operationally relevant. Each interval corresponds to a critical phase of the pandemic:

- 1 March 2020: Captures the early stages of the pandemic, including the first wave of infections before large-scale public health interventions and before the emergence of COVID-19 variants.
- 1 July 2020: Reflects the summer wave, caused by reopening policies and a resurgence of cases.
- 1 November 2020: Captures the major winter surge, characterized by the highest infection peaks before vaccine distribution.
- 1 April 2021: Corresponds to the post-vaccine introduction period and the rise of the Alpha variant.
- 1 November 2021: Represents the period dominated by the Delta variant and the emergence of Omicron, with renewed public health responses such as booster campaigns and travel restrictions.

These intervals allow us to assess how each model performs across distinct phases of the pandemic, capturing shifts in transmission dynamics, public health interventions, and viral characteristics.

To ensure fair comparisons among the models, we calibrated each model’s parameters to achieve a basic reproduction number R0 within the range of [1.2, 1.6], reflecting the average transmission rates observed during different pandemic phases [40]. The basic reproduction number, R0, represents the average number of individuals infected by a single case in a susceptible population. An R0≥1 indicates that the disease is spreading exponentially. In Appendix A, we provide the specific values for each model’s calibrated parameters. The SEAIRD, however, optimizes its parameters based on the data, and as a result, its reproduction may deviate from the other models.

Prior studies have shown that COVID-19 unfolded in broadly similar epidemic phases, which are typically marked by exponential growth, peaking, and decline across different national contexts. For example, ref. [41] demonstrated that epidemic trajectories across North, Central, and South American countries were largely synchronized, particularly during the initial waves of the pandemic. These findings suggest that intervention timing and transmission dynamics, rather than country-specific healthcare infrastructure, play a dominant role in shaping outbreak curves. Our selected five-phase U.S. timeline effectively reflects this broader pattern, thereby offering a representative framework for comparing model performance. In Section 4.5, we further complement this design by assessing robustness to key parameter variation, specifically vaccination coverage, hospitalization, and transmission rates.

## 4. Results and Discussions

In this section, we present our main findings and provide managerial insights for healthcare decision makers.

### 4.1. Model Performance Evaluation

In this section, we evaluate the predictive accuracy. While the term “accuracy” is often associated with machine learning and predictive models (e.g., classification), in the context of compartmental epidemic models, it generally refers to the goodness of fit between predicted and observed infection trends. This is typically quantified using error-based metrics such as RMSE or MAE, where lower values indicate better model fit. In this study, we evaluate the performance of four compartmental models, SIR, SEIRD, SEAIRD, and SIDARTHE across various pandemic phases to assess their predictive capability. The analysis is divided into three main components: (1) an integrated assessment of model performance using both qualitative insights and quantitative Root Mean Square Error (RMSE) evaluations; (2) an analysis of prediction orientation, focusing on whether each model tends to over- or under-predict infection numbers; (3) and an evaluation of the accuracy of predicting the time to peak infections. By comparing these models, we aim to understand how different assumptions, complexities, and parameter dynamics affect their ability to forecast COVID-19 infection trends and inform healthcare resource allocation.

#### 4.1.1. Model Performance Across Pandemic Phases

**Figure 2:**
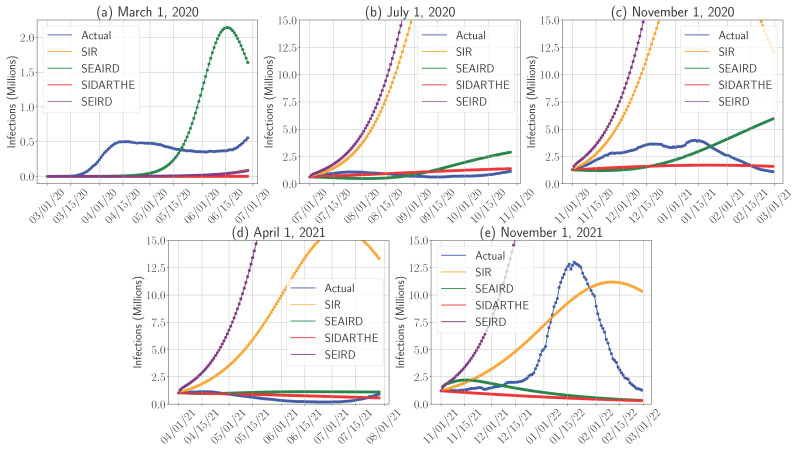
Comparison of actual and predicted daily COVID-19 infections in the U.S. for the SIR, SEIRD, SEAIRD, and SIDARTHE models.

**Figure 3:**
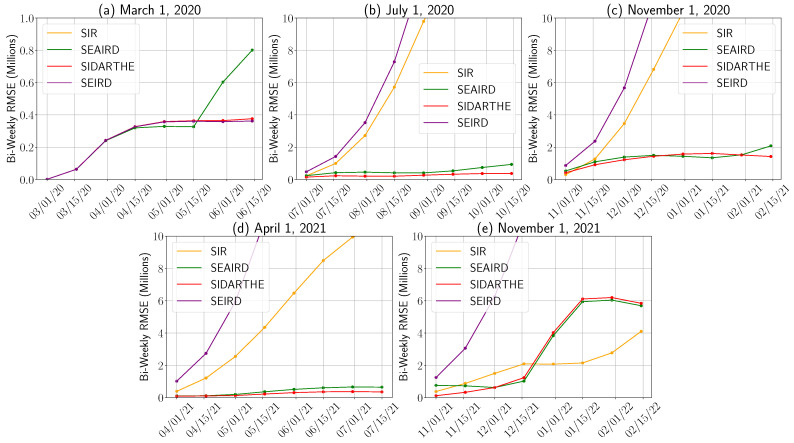
Bi-weekly RMSE for the SIR, SEIRD, SEAIRD, and SIDARTHE models.

**Figure A1:**
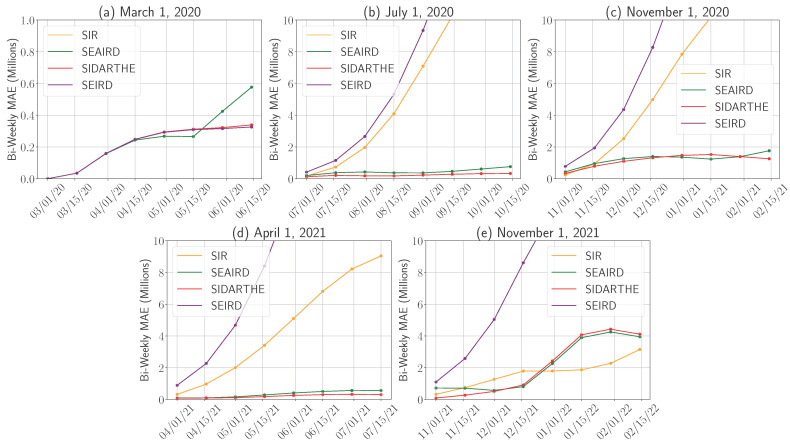
Bi-weekly MAE for the SIR, SEIRD, SEAIRD, and SIDARTHE models.

In the following analysis, we simulate disease progression based on the calibrated parameters and initial conditions for each chosen date, covering 120-day intervals. Given that some models do not differentiate between symptomatic and asymptomatic cases, we first focus on the total number of infections (combining both groups), as shown in Figure 2. The RMSE, presented in Figure 3, is computed bi-weekly (as an average of a 15-day period) to measure prediction accuracy. Lower RMSE values indicate better alignment between predicted and actual infection numbers. RMSE helps in quantifying the model’s performance over time, allowing us to see how well each model adapts to different phases of the pandemic. We also refer readers to Figure A1 in Appendix C, which presents the mean absolute error (MAE) of each model across all periods, noting that the MAE results follow a similar trend to those observed with the RMSE metric. Understanding the total number of infections is important for healthcare policymakers, as it reflects the overall burden on the healthcare system. Several key insights, supported by RMSE values, emerge from these simulations.

First, in the early stages of the pandemic (1 March 2020), all models struggled to accurately predict infection numbers due to data scarcity and uncertainties around human interaction patterns. The small number of infections relative to the large susceptible population presented additional challenges, as small changes in initial infection levels could lead to significant variations in the predicted trajectories. This sensitivity to initial conditions is especially pronounced in models like SEAIRD, which incorporates asymptomatic carriers and attempts to optimize numerous parameters. The SEAIRD model overestimated infections, peaking at around 2 million cases by mid-May, leading to a sharp rise in RMSE during this period. The model’s complexity, combined with limited early data, likely resulted in overfitting and inflated predictions. In contrast, SIDARTHE, SIR, and SEIRD consistently underestimated infections, but their lower RMSE values reflect a more conservative prediction pattern, which did not diverge as drastically from actual numbers. However, their delayed response to infection growth and simpler structures (e.g., excluding asymptomatic compartments in SIR and SEIRD) contributed to their inability to capture the rapid rise in cases that began around mid-April.

Second, while the summer wave (1 July 2020), the winter surge (1 November 2020), and the post-vaccine introduction period (1 April 2021) share some similar model dynamics, each period reveals distinct differences in performance when compared to actual infection patterns. During the summer wave, infections rose gradually, peaking in late July with a relatively mild increase. SIR and SEIRD consistently overestimated the number of cases, forecasting much sharper rises than observed. This overestimation is reflected in their higher RMSE values, which indicate difficulty in capturing the slower, more gradual infection trends seen during this period. In contrast, SEAIRD and SIDARTHE, which incorporate asymptomatic carriers and other compartments, produced more conservative predictions, and hence lower RMSE values. Their complex modeling of disease transmission allowed them to capture the slower dynamics more accurately. The winter surge, which peaked between mid-December and mid-January, revealed even more pronounced differences among the models. SIR and SEIRD again over-predicted infections, with RMSE values rising accordingly. Their simpler structures could not account for the complex transmission dynamics during this period of increased social interaction. Meanwhile, SEAIRD underestimated the speed of the surge but outperformed SIR and SEIRD, showing lower and more stable RMSE values. SIDARTHE failed to predict a peak, consistently underestimating actual infections, yet still demonstrated better overall accuracy compared to the simpler models. In the post-vaccine period, none of the models explicitly accounted for vaccination, limiting their ability to accurately capture this phase. SIR and SEIRD continued to overestimate infections, leading to higher RMSE values, while SEAIRD and SIDARTHE, though not directly modeling vaccination, exhibited lower RMSE values by accounting for both symptomatic and asymptomatic spread.

**Figure 4:**
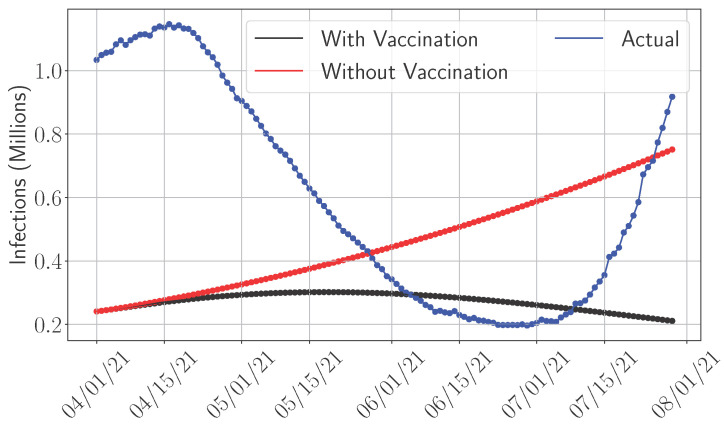
Impact of vaccination on SIDARTHE accuracy post-vaccination, i.e., post 1 April 2021.

Given that vaccination significantly reduces actual infections, and all models tended to overestimate infections during the post-vaccination period (after 1 April 2021, as shown in Figure 2d), we examined the effect of incorporating a vaccination policy into the compartmental framework. Specifically, we introduced a vaccination rate η into the model to assess its impact on predictive accuracy. Since SIR and SEIRD models substantially over-predict and the more accurate SEAIRD model is adaptive and computationally intensive due to its training requirements, we focus this analysis on the SIDARTHE model. Based on reported data [42,43], the average daily number of vaccine doses administered in the U.S. was approximately 641,198, corresponding to a rate of η=0.0025. We also account for the possibility of reinfection, allowing recovered individuals to return to the susceptible class at a rate $\hat{r}$, reflecting immunity loss observed in COVID-19 cases after roughly four months [29], which translates to $\hat{r}=\frac{1}{120}$. The adjusted SIDARTHE model is detailed in Appendix B. Figure 4 clearly states that incorporating vaccination and reinfection, with vaccination having the dominant effect, leads to a noticeable improvement in approximating actual infection trends. This modification results in an 11% increase in predictive accuracy, as measured by the mean absolute error.

Third, continuing on Figure 2, during the period marked by the dominance of the Delta variant and the emergence of Omicron (beginning 1 November 2021), infection levels peaked around mid-January 2022. During this phase, the SIR model performed notably well in capturing the second peak, suggesting that even relatively simple models can remain effective during periods of rapid case growth. The model also achieved relatively low RMSE values, indicating its ability to closely approximate the overall infection trend. This makes it particularly valuable for long-term planning efforts, such as forecasting the demand for hospital beds and intensive care units. However, even the SIR model slightly underestimated the magnitude of the peak, as reflected in its RMSE values, showing its limitations in capturing the full extent of infections during phases with rapidly changing transmission rates. SEAIRD and SIDARTHE, which include asymptomatic compartments, struggled more during this period, with rising RMSE values indicating their difficulty in keeping pace with the rapid spread of the Delta and Omicron variants. SEIRD, in particular, continued to overestimate infections significantly, showing higher RMSE values and diverging further from actual trends.

*Simpler models like SIR often perform well during high infection peaks, such as those seen with Delta and Omicron, by capturing broad trends. In contrast, complex models like SEAIRD and SIDARTHE better reflect gradual spread through asymptomatic transmission but struggle with rapid surges, as shown by higher RMSE values. Incorporating vaccination into these models can improve accuracy, especially once vaccines become available. These findings highlight the value of hybrid approaches that combine the robustness of simple models with the flexibility of complex ones. While this section focused on infection prediction, understanding whether models tend to over- or under-predict is also critical for guiding resource allocation, which we explore next.*

#### 4.1.2. Prediction Orientation: Over- and Underestimation Analysis

In this section, we explore the orientation of each model’s predictions and whether they consistently overestimate or underestimate actual COVID-19 cases. An overestimation occurs when the compartmental model predicts values higher than the actual observed data, whereas an underestimation refers to predictions that fall below the actual values. Overestimations may lead to unnecessary resource allocation, causing a surplus in some areas and potential shortages in others. Conversely, underestimations, especially during peak infection periods, might result in excessive demand, which can overwhelm healthcare systems.

**Figure 5:**
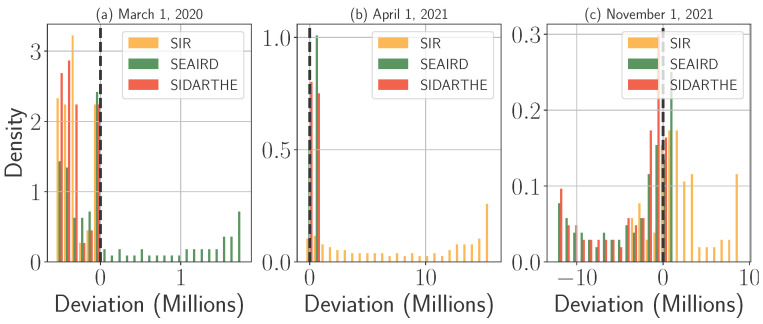
Distribution of deviation from actual COVID-19 daily data in the U.S. for the dates 1 March 2020, 1 April 2021, and 1 November 2021.

To explore this further, Figure 5 illustrates the deviation between predicted and actual infections, defined as deviation=predicted−actual. Positive deviations indicate over-predictions, while negative deviations reflect under-predictions. In other words, densities where deviations are positive (greater than 0) indicate the frequency of overestimations, while densities where deviations are negative (less than 0) represent underestimations. In addition, we present in Table 4 a summary of the histogram results in which we report the average proportion of days in each month during which each model over- or underestimated the actual infection numbers, along with the corresponding average monthly deviations. Our analysis focuses on three models, SIR, SEAIRD, and SIDARTHE, across three critical pandemic stages: early pandemic (1 March 2020), post-vaccine introduction (1 April 2021), and the Delta/Omicron phase (1 November 2021). SEIRD is omitted from this analysis due to its consistently low accuracy beyond the early stages of the pandemic. The other models demonstrated higher accuracy when the number of cases became large significant.

During the early phase of the pandemic, all models exhibit a strong tendency toward under-prediction, as seen in the negative deviations in Figure 3a. This result is also clear in Table 4 (first four rows), where deviations are all negative for all models and proportions of overestimations are, for the majority of the cases, 0. This is especially evident in the SIR and SIDARTHE models, which consistently underestimate case numbers. The scarcity of reliable data, combined with rapidly shifting human behavior, made it difficult for these simpler models to capture the initial explosive spread of the virus. SEAIRD, in contrast, dynamically adjusts its parameters based on limited data, leading to over-prediction in this phase. While its flexibility can be advantageous in later stages, SEAIRD’s tendency to overfit early data resulted in inflated infection estimates. This over-prediction likely led to the over-preparation of resources, whereas the under-predictions from SIR and SIDARTHE may have left healthcare systems underprepared for surges in moderate case rises.

By the post-vaccine period, the prediction orientation of all models shifts toward over-prediction. As shown in Figure 3b, SEAIRD and SIDARTHE display smaller errors than earlier phases, suggesting that their predictive accuracy improved as more data became available. Table 4 (second four rows) confirms this observation, where deviations are for the majority of the models positive, and proportions of overestimations are high. However, both SIR and SIDARTHE, which previously under-predicted infections, now over-predict case numbers, likely reflecting the uncertainty introduced by vaccination efforts and changing virus transmission dynamics. Despite its dynamic parameter optimization, SEAIRD still struggles to account for the growing vaccinated population, leading to slight over-prediction. These errors suggest that failing to account for vaccination effects caused all models to overestimate transmission rates, resulting in potential over-preparation of resources during a period of declining infections.

As illustrated in Figure 3c, the prediction dynamics shift notably during the surge driven by the Delta and Omicron variants. While the SIR model generally overestimates infections before and after the peak, it comes closest to capturing the timing and shape of the major wave observed in mid-January 2022. However, it still slightly underestimates the peak itself, reflecting limitations in accounting for the accelerated transmission rates associated with these highly contagious variants. This behavior is further supported by the average deviations and overestimation proportions reported in the final four rows of Table 4. SIDARTHE and SEAIRD continue to exhibit under-prediction tendencies, particularly as they fail to keep pace with the fast-evolving dynamics of the Delta and Omicron variants. In the case of SEAIRD, this underperformance can be attributed to its parameter optimization on pre-variant data, making it less responsive to the sharp infection rise during the variant-driven surge. This is also clear in Table 4 by looking at SEAIRD negative deviations and low proportions of overestimations. This underestimation may have led to resource shortages during a critical period of heightened hospital admissions.

*The deviation analysis highlights how each model behaves across different pandemic stages. Early on, SIR and SIDARTHE tend to under-predict, risking under-preparedness, while SEAIRD often over-predicts due to overfitting, potentially leading to resource oversupply. In the post-vaccine phase, all models overestimate infections, struggling to capture vaccination effects. During the Delta/Omicron wave, SIR aligns best with peak trends, while SEAIRD and SIDARTHE under-predict the rapid spread. For policymakers, understanding these tendencies is key. Flexible models like SEAIRD may be valuable for early-stage, worst-case planning, while simpler models like SIR can better capture peak trends for long-term decisions. No single model, however, fully captures the dynamics of fast-evolving variants. Adopting hybrid approaches, combining the strengths of different models, could lead to more balanced and effective pandemic management strategies. The design of such hybrids should consider factors such as the stage of the pandemic, data availability, and whether short-term responsiveness or long-term interpretability is prioritized. We expand on this in Section 4.4 with examples and further guidance.*

#### 4.1.3. Time to Peak Prediction Error

An additional metric for evaluating the prediction accuracy of compartmental models is the error in forecasting the timing of the infection peak. This has important practical implications, particularly for healthcare resource planning. A small timing error allows agencies to better allocate critical resources such as hospital beds and intensive care units during periods of peak demand. Table 5 reports the absolute difference in days between the predicted and actual peak times for each model across all time periods. The results show that during the early and middle stages of the pandemic, up to 1 April 2020, most models predicted peak timing accurately, with SEAIRD showing the lowest error. After 1 April 2020, prediction errors increased, likely due to the widespread rollout of vaccination campaigns in the United States, with the SIDARTHE model providing the most accurate peak timing estimates during this phase.

### 4.2. Models’ Predictability for Symptomatic and Asymptomatic Infections

**Figure 6:**
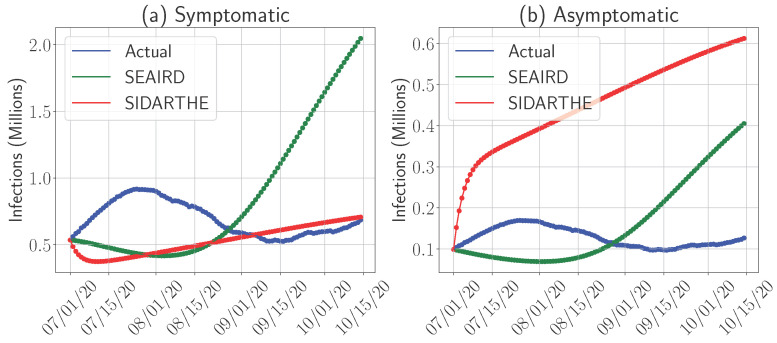
Comparison of actual and predicted numbers of symptomatic and asymptomatic infections for the SEAIRD and SIDARTHE models, starting from 1 July 2020.

Given the importance of distinguishing between symptomatic and asymptomatic carriers for both healthcare resource planning and controlling virus transmission, we now turn our attention to assessing the performance of the SEAIRD and SIDARTHE models, which are specifically designed to account for both symptomatic and asymptomatic cases. These two compartments, which collectively define the total infected population, play critical roles in understanding and managing pandemic dynamics. Figure 6 presents the actual versus predicted numbers of symptomatic cases (Figure 6a) and asymptomatic cases (Figure 6b) using both models for the 1 July 2020 period.

Accurately predicting symptomatic infections is essential for the allocation of critical healthcare resources, such as ventilators, ICU beds, and hospital staff. Symptomatic individuals, especially those with severe symptoms, often require immediate medical attention, and failure to accurately forecast these numbers could lead to resource shortages and increased mortality. On the other hand, predicting asymptomatic infections is vital for controlling virus transmission, as asymptomatic carriers unknowingly spread the virus through interactions with others. Accurate predictions of asymptomatic cases are key for designing effective mass screening policies and ensuring that adequate testing resources are available to identify and isolate asymptomatic carriers before they further spread the virus [35].

The performance of the SEAIRD and SIDARTHE models in predicting these two categories reveals important trade-offs:

- **Predicting Symptomatic Cases:** As shown in Figure 6a, the SIDARTHE model demonstrates better accuracy in predicting the number of symptomatic cases compared to SEAIRD. SIDARTHE’s calibrated approach allows it to capture the established dynamics of symptomatic infections, which are easier to track due to observable symptoms and subsequent quarantine or hospitalization.
- **Predicting Asymptomatic Cases:** Conversely, Figure 6b shows that the SEAIRD model performs better in predicting the number of asymptomatic cases. Since asymptomatic individuals do not self-quarantine or seek treatment, their movement and interaction patterns are harder to track and model. SEAIRD’s dynamic parameter optimization allows it to better adapt to these complex dynamics. However, this adaptability comes with a trade-off: SEAIRD tends to overestimate symptomatic cases, as seen in Figure 6a, likely due to the focus on capturing asymptomatic dynamics.

For policymakers, understanding these trade-offs is critical for making informed decisions about resource allocation and intervention strategies. When the healthcare system’s priority is managing severe symptomatic cases, the SIDARTHE model may offer better guidance, as it provides more accurate forecasts of symptomatic infections. On the other hand, when the primary objective is to contain the spread of the virus and reduce pressure on healthcare capacity, especially during phases with high asymptomatic transmission, the SEAIRD model’s strength in predicting asymptomatic cases becomes highly valuable. Asymptomatic carriers are major drivers of transmission, and SEAIRD’s predictions could assist public health officials in deploying targeted mass testing or screening campaigns. SEAIRD’s ability to capture asymptomatic spread also offers essential insights for designing quarantine and isolation strategies, supporting efforts to contain the virus early.

The contrasting performance of the SEAIRD and SIDARTHE models highlights a broader trade-off in pandemic modeling: balancing the need for accurate predictions of both symptomatic and asymptomatic cases. However, models like SEAIRD, which accurately capture asymptomatic spread through dynamic parameter optimization, may overestimate symptomatic cases, while SIDARTHE’s strength in predicting symptomatic infections can lead to underestimation of asymptomatic carriers. From a strategic perspective, these differences underscore the importance of selecting the right model based on the pandemic stage and specific goals. In the early stages of a wave, when asymptomatic transmission drives the spread, SEAIRD’s insights are particularly valuable for informing mass testing and isolation policies. As the focus shifts toward managing severe cases, SIDARTHE’s strength in predicting symptomatic infections becomes critical for resource allocation in intensive care settings.

### 4.3. Summary of Compartmental Model Evaluation

Table 6 provides a comparative summary of compartmental model performance across various evaluation metrics and pandemic phases. The results show that model effectiveness depends heavily on both the metric used and the stage of the outbreak. For example, while SEAIRD performs well in terms of RMSE and MAE during the mid phase, it falls short in predicting the time to peak. In contrast, SIDARTHE shows stronger performance in estimation orientation and accuracy during the later phase. These variations indicate that no single model is optimal in all scenarios. Therefore, model selection should align with the priorities of the healthcare decision maker, whether the focus is on minimizing prediction error, anticipating peak timing, or balancing overestimation and underestimation. It is also important to consider the planning time frame. Models with better short-term accuracy support the timely allocation of short-term resources, while those with better long-term accuracy are more suitable for planning for long-term resources. Additionally, including pandemic-related factors such as lockdowns, testing, and vaccination can further improve model accuracy. For instance, Figure 4 shows that adding a vaccination component to the SIDARTHE model improves predictive accuracy by approximately 11%. In the following section, we enhance the nonadaptive models into adaptive ones, focusing on SEAIRD, and show that adaptive models achieve higher short-term predictive accuracy.

### 4.4. Adaptive Versus Non-Adaptive Models

**Figure 7:**
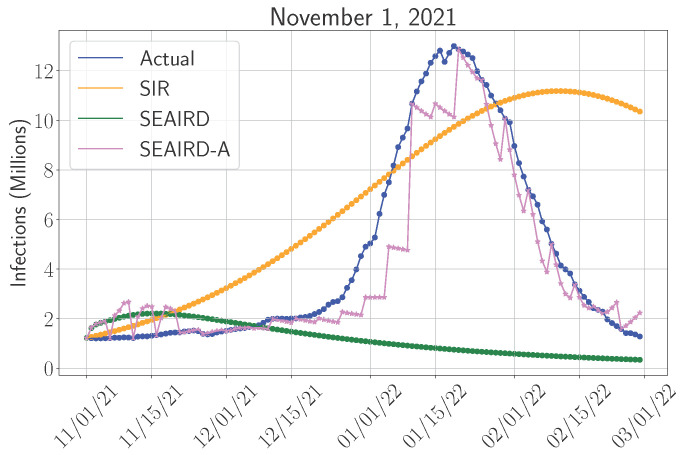
Predicted number of infections using the adaptive SEAIRD model (SEAIRD-A) compared to the non-adaptive model, starting from 1 November 2021.

Figure 7 compares the performance of the SIR model, a non-adaptive SEAIRD model, and an adaptive SEAIRD model (denoted as SEAIRD-A) for the 1 November 2021 period, dominated by the Delta variant and the rapid emergence of Omicron. This period represents one of the most challenging phases of the pandemic, marked by rapid transmission and a sharp increase in cases. SIR was selected for this comparison because it performed best for this period among the simpler models, while SIDARTHE was excluded due to its lower predictive accuracy during this phase. In this context, it is essential to assess how adaptive models that can dynamically recalibrate parameters compared to traditional, static approaches.

The non-adaptive SEAIRD model optimizes its parameters once, based on early data, and then predicts the entire 120-day period. This approach, while computationally efficient, is unable to capture the rapid changes in transmission dynamics that occur during periods of variant-driven surges. As seen in Figure 7, the non-adaptive SEAIRD model (green line) severely underestimates infections, particularly during the sharp rise in cases caused by Omicron. This underperformance is largely due to the fact that the model’s parameters were optimized based on data before the infection peak. As such, the model was not flexible enough to adjust to the sudden increase in transmission rate that characterized this period.

This illustrates a key limitation of non-adaptive models: while they offer stability and are computationally less demanding, they are often too rigid to effectively handle scenarios where pandemic dynamics change rapidly. This becomes problematic when decision-makers need up-to-date insights for immediate resource allocation.

In contrast, the adaptive SEAIRD-A model (pink line) recalibrates its parameters every five days, allowing it to continually adjust its predictions in response to real-time data. As shown in Figure 7, SEAIRD-A closely tracks the actual infection trend, peaking at nearly the same time and magnitude as the real data. This short-term accuracy is important during periods of rapid viral spread, where public health systems must quickly adjust resource allocation.

The ability to continuously optimize parameters gives the adaptive model a clear advantage in providing timely insights for immediate action. However, there are trade-offs. Adaptive models, by design, require frequent recalibration, which comes with increased computational costs and longer runtime. Additionally, adaptive models are most effective when the decision-making horizon is short, such as days or weeks, where the focus is on allocating resources that can be quickly mobilized.

While the adaptive SEAIRD-A model offers superior short-term accuracy, its reliance on frequent recalibration makes it less suited for long-term strategic planning. For instance, in scenarios where healthcare policymakers need to prepare for resources that have longer lead times, such as acquiring ventilators, constructing new facilities, or scaling up permanent hospital capacity, non-adaptive models like SEAIRD or SIR may provide more actionable insights. These models, despite being less accurate in predicting the exact peak of infections, offer a broader view of pandemic trends over a longer period. One key advantage of non-adaptive models is their ability to provide early warnings. For example, even if such a model projects a slower rise in cases, it can still give policymakers enough time to begin preparations for long-term resource needs well before the infection peak is reached.

The SIR model (orange line) is a prime example of a non-adaptive model that performs well on average during periods of high infection peaks. As seen in Figure 7, SIR slightly underestimates the peak but offers a reasonable approximation of the overall trend. This makes it particularly valuable for long-term decision-making, where policymakers need to anticipate the scale of a surge months in advance and make strategic preparations accordingly. In such cases, SIR’s ability to predict the general shape of the infection curve, even if it is not perfectly aligned with real-time data, makes it a useful tool for planning long-term resource allocation.

On the other hand, adaptive models like SEAIRD-A are best suited for situations that require flexibility. For instance, once a surge in infections has already begun, an adaptive model can provide real-time updates, allowing decision-makers to fine-tune resource allocation on a day-to-day basis. This might include reallocating existing hospital beds, deploying staff to areas experiencing high case loads, or determining where additional testing or vaccination efforts are most urgently needed.

To maximize preparedness and response, decision-makers may benefit from adopting a hybrid approach that leverages the strengths of both adaptive and non-adaptive models. For example, non-adaptive models like SIR can support long-term strategic planning, while adaptive models like SEAIRD-A are better suited for short-term, data-driven decisions in rapidly evolving contexts. This complementary use has proven useful for challenges such as managing ICU capacity or allocating newly arrived vaccine shipments.

Designing such hybrid models requires the careful consideration of several factors. First, the stage of the epidemic plays a critical role: early exponential growth phases may benefit from more flexible, data-driven models, while later, plateauing stages might be better captured by mechanistic models with saturation dynamics. Second, the forecasting horizon and decision context matter; that is, short-term operational decisions (e.g., ICU allocation) may require models that adapt quickly to new data, whereas long-term strategic planning (e.g., infrastructure expansion) favors stability and interpretability. Third, the availability and reliability of input data should guide hybrid construction: high-quality real-time data support more adaptive elements, while data-poor environments may necessitate more structured components.

**Figure 8:**
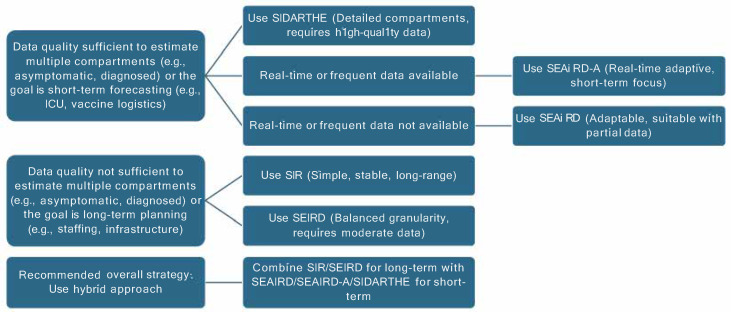
Decision tree for selecting epidemic forecasting models based on data availability, epidemic stage, and forecasting goals.

Finally, the complementary strengths and error structures of the candidate models should be evaluated; hybridization is most effective when models offer orthogonal strengths, such as one model capturing peak timing well and another minimizing cumulative bias. For example, simpler models such as SIR or SEIRD can provide stable long-range forecasts, while adaptive models like SEAIRD and SEAIRD-A offer greater responsiveness, and detailed models like SIDARTHE capture nuanced compartmental dynamics but require high-quality data and intensive parameter estimation. Overall, a hybrid approach that combines the long-term stability of simpler models with the short-term adaptability of more complex models is recommended. These criteria are summarized in Figure 8, which provides a practical decision tree to guide model selection based on data availability, epidemic stage, and forecasting objectives.

*In conclusion, while the adaptive SEAIRD-A model outperforms non-adaptive models in terms of short-term accuracy, the choice between adaptive and non-adaptive approaches depends on the specific decision-making context. For long-term planning, non-adaptive models, despite their less precise forecasts, offer the benefit of early warnings and stability, which are critical for preparing resources with long lead times. For short-term, real-time responses, adaptive models provide the flexibility needed to adjust to rapidly changing conditions, ensuring that resources are deployed when and where they are needed most.*

### 4.5. Sensitivity Analysis

In this section, we shift our focus to examining the impact of key parameters that influence disease dynamics. We select the SIDARTHE model for this analysis due to its accuracy and relatively low computational complexity and the absence of training requirements, in contrast to the SEAIRD model. Our study specifically considers the time period from 1 November 2020 (we chose this date as the vaccine became available in December 2020 [44]) to 15 February 2021. Although the primary objective of this work is to compare the predictive accuracy of various models, this section broadens the scope by exploring the influence of several important epidemiological factors. **First**, given the adjustment of the SIDARTHE model to incorporate the vaccination campaigns (see Section 4.1.1 and the formulation in Appendix B), we analyze the impact of vaccination by varying the vaccination rate and measuring its influence on total infection counts. **Second**, we assess the potential effects of hospitalization by varying the recovery rate. This is motivated by the assumption that individuals under medical care—such as those in intensive care units—may recover more quickly. In addition, such a study can also reflect the importance of the healthcare capacity, which can accommodate more patients; higher recovery rates may result from improved care or increased hospital capacity. **Third**, we measure the role of the transmission rate on the disease progression. The transmission rate reflects the population’s behavioral response to interventions such as lockdowns and social distancing, and its variation provides insight into the effectiveness of such policies in reducing infection spread.

**Figure 9:**
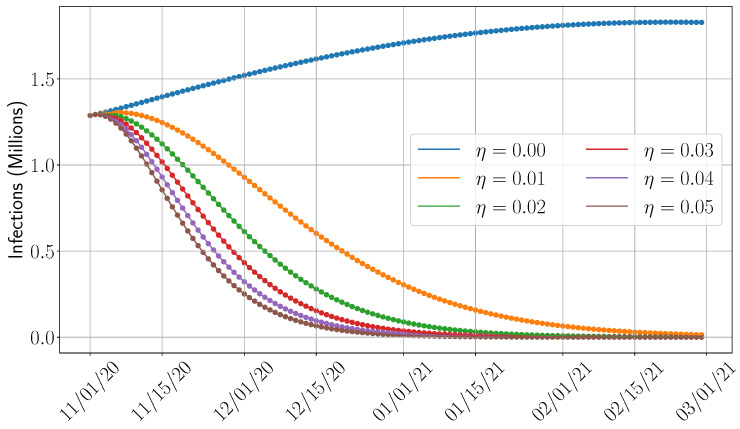
Impact of vaccination on the total infections.

In Figure 9, we illustrate the variation in total infections under six different vaccination rates η. As expected, increasing vaccine availability leads to a substantial reduction in infections. For instance, at a vaccination rate of 1%, the total number of infections decreases by approximately 70%. Note that with higher rates of vaccination (e.g., 5%), peak infections can be avoided, which can significantly reduce the pressure on the healthcare system.

**Figure 10:**
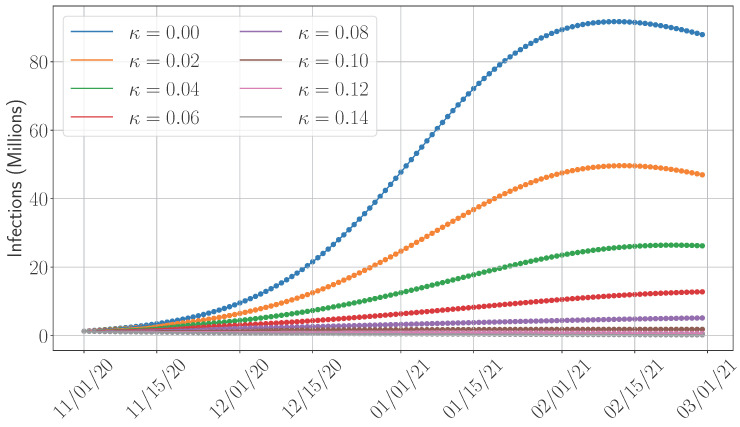
Impact of the hospitalization rate on the total infections.

Figure 10 examines the impact of hospitalization rates on the number of infections, modeled through variations in the recovery rate $\gamma^{T}$ of threatened individuals, i.e., those in compartment T, which experience severe symptoms and require hospitalization. While the effect is less pronounced compared to vaccination, increased hospitalization capacity can still contribute to a meaningful reduction in infections—and consequently, in mortality rates. For example, a 2% increase in the hospitalization rate results in an approximately 46% decrease in total infections.

**Figure 11:**
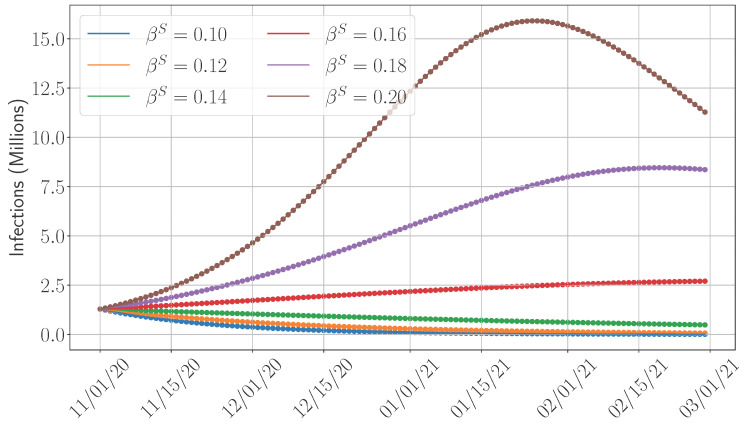
Impact of the transmission rate on the total infections.

We now examine the impact of probably the most crucial parameters that fundamentally control the disease progression, namely the transmission rate $\beta^{S}$ of symptomatic individuals. To that end, we plot in Figure 11 the total number of infections for different values of $\beta^{S}$. The impact is pronounced; being able to reduce the transmission rate by 20% can drastically reduce infections by about 60%. Finally, we would like to emphasize that coordinated public health measures, such as timely vaccination campaigns, implementation of lockdowns and social distancing, and expanding hospitalization capacity, can have a profound impact on mitigating disease spread and reducing pressure on the healthcare system.

We note that the incorporation of vaccination, reinfection, and hospitalization increases the flexibility of a compartmental model and supports its application to future infectious disease outbreaks beyond COVID-19. With the appropriate calibration of parameters, especially the basic reproduction number $R_{0}$, the model can capture the dynamics of a wide range of emerging diseases.

## 5. Conclusions

In this paper, we provide a brief overview of the pandemic forecasting literature and evaluate the predictive accuracy of several compartmental epidemic models, including SIR, SEIRD, SEAIRD, and SIDARTHE, by applying them to real-world COVID-19 data. Our primary objective is to assess how the complexity of each model influences its ability to forecast disease progression and guide resource allocation. To achieve this, we first discuss the main takeaways of this study, followed by an examination of its limitations and potential directions for future research.

### 5.1. Summary of Insights Gained and Further Discussion

The analysis reveals that while more complex models, such as SEAIRD and SIDARTHE, offer deeper insights into disease dynamics, simpler models like SIR can outperform these more intricate models under specific conditions, particularly during the early stages of the pandemic or when the focus is on predicting peak infection trends.

A key insight from this study is that model complexity does not necessarily guarantee better predictions. While models with more compartments capture a wider range of epidemiological dynamics, such as distinguishing between symptomatic and asymptomatic individuals, they also increase the risk of overfitting when data is limited. For example, the SEAIRD model, which incorporates asymptomatic carriers and optimizes parameters, tended to overestimate infections during the early stages due to its sensitivity to limited initial data.

In contrast, simpler models like SIR proved to be more robust when long-term trends were the focus, particularly for forecasting peak infection periods. This has practical implications for healthcare policymakers, as models like SIR can support strategic planning by predicting the trajectory of the pandemic without overreacting to short-term fluctuations. On the other hand, adaptive models like SEAIRD provide valuable short-term insights and are particularly useful for guiding rapid interventions, such as testing, screening, or allocating limited healthcare resources.

Additionally, the trade-offs between predicting symptomatic and asymptomatic infections highlight the importance of a balanced approach to resource allocation. While SEAIRD is more accurate in predicting asymptomatic spread, SIDARTHE performs better when it comes to forecasting symptomatic cases, which are critical for planning ICU capacity and ventilator needs. By combining insights from both models, healthcare planners can more effectively manage the pandemic, gaining better guidance on controlling transmission while simultaneously addressing severe cases.

### 5.2. Limitations and Future Work

Although this study provides important insights, it is subject to certain limitations that also offer opportunities for future research. First, data constraints led to the exclusion of some compartments, such as hospitalization and testing-related compartments, which might have provided further insight into the impact of healthcare capacity. Future work could integrate more detailed datasets to improve predictions, particularly in relation to healthcare system constraints.

Another limitation is that most of the models assume relatively static parameters, except for the adaptive SEAIRD model. In real-world scenarios, however, factors such as public health measures, changes in human behavior, and the emergence of new variants are constantly evolving. Future models should incorporate real-time data streams to allow for continuous recalibration of parameters, better reflecting the dynamic nature of a pandemic’s progression.

Further research could explore hybrid models that combine the simplicity and stability of SIR for long-term forecasting with the adaptability of SEAIRD or SIDARTHE for short-term interventions. This would enable policymakers to make more informed decisions about resource allocation over both immediate and extended time horizons. Additionally, expanding epidemic models to consider economic and social impacts, such as the economic costs of lockdowns and the influence of public health measures on social behavior, would provide a more comprehensive tool for weighing healthcare outcomes alongside broader societal and economic considerations. Finally, as new viral variants emerge, future models should account for evolving vaccine efficacy and the potential for more transmissible variants, leading to better predictions about the progression of pandemics and helping governments prepare more effectively for future pandemic waves.

## Figures and Tables

**Table 1:** Summary of compartmental models discussed in the literature, including their compartments, key features, application examples, and limitations.

Model	Compartments	Key Feature	Application Example	Limitations
SIR	S, I, R	Simple baseline model	[4,18]	Ignores exposed/asymptomatic states
SEIR	S, E, I, R	Adds incubation (latent) period	[24,25]	No explicit asymptomatic differentiation
SEIRD	S, E, I, R, D	Tracks mortality explicitly	[30]	Assumes permanent immunity
SIQRD	S, I, Q, R, D	Models quarantine for false positives	[35]	Requires detailed testing/quarantine data
SEAIRD	S, E, A, I, R, D	Includes asymptomatic spread dynamics	[9,32]	Needs asymptomatic prevalence data
SIDARTHE	S, I, D, A, R, T, H, E	High compartmental granularity (diagnosis stages)	[10]	High data and parameter demands

**Table 2:** Mathematical models and corresponding parameters.

Model	Equations	Parameters
**SIR**	$\frac{dS}{dt}=-\frac{\beta SI}{N}$, $\frac{dI}{dt}=\frac{\beta SI}{N}-\gamma I$, $\frac{dR}{dt}=\gamma I$	β: Rate of transmission. γ: Rate of recovery.
**SEIRD**	$\frac{dS}{dt}=-\frac{\tilde{\beta }SI}{N}+\alpha E,$ $\frac{dE}{dt}=\frac{\tilde{\beta }SI}{N}-\rho E-\alpha E,$ $\frac{dI}{dt}=\rho E-\left(\gamma +\delta \right)I,$ $\frac{dR}{dt}=\gamma I,$ $\frac{dD}{dt}=\delta I$	$\tilde{\beta }$: Rate of exposure to infected individuals. α: Probability of incubated individuals turning negative. ρ: Rate of incubated individuals turning infectious. δ: Rate of mortality.
**SEAIRD**	$\frac{dS}{dt}=-\frac{\phi \bar{\beta }S\left(\epsilon E+I+A\right)}{N}+\alpha E+\xi R,$ $\frac{dE}{dt}=\frac{\phi \bar{\beta }S\left(\epsilon E+I+A\right)}{N}-\rho E-\alpha E,$ $\frac{dA}{dt}=\left(1-\theta \right)\rho E-\left(\gamma +\delta \right)A,$ $\frac{dI}{dt}=\theta \rho E-\left(\gamma +\delta \right)I+\nu R,$ $\frac{dR}{dt}=\gamma \left(I+A\right)-\left(\nu +\delta +\xi \right)R,$ $\frac{dD}{dt}=\delta \left(I+A+R\right)$	$\bar{\beta }$: Rate of exposure to incubated individuals. ϕ: Probability of exposure to infection. ξ: Ratio of reverting to the susceptible after recovery. θ: Fraction of exposed individuals becoming symptomatic. ν: Rate of cured individuals turning positive.
**SIDARTHE**	$\frac{dS}{dt}=\frac{-S(\beta^{S}I+\beta^{\mathrm{AS}}A)}{N},$ $\frac{dI}{dt}=\zeta A-\left(\mu +\gamma^{S}\right)I,$ $\frac{dA}{dt}=\frac{S(\beta^{S}I+\beta^{\mathrm{AS}}A)}{N}-\left(\gamma^{\mathrm{AS}}+\zeta \right)A$ $\frac{dT}{dt}=\mu I-\left(\delta +\gamma^{T}\right)T,$ $\frac{dH}{dt}=\gamma^{S}I+\gamma^{\mathrm{AS}}A+\gamma^{T}T,$ $\frac{dE}{dt}=\delta T.$	$\beta^{S}$ and $\beta^{\mathrm{AS}}$: Rates of transmission of symptomatic and asymptomatic individuals, respectively. ζ: Rate of asymptomatic individuals developing symptoms. μ: Fraction of symptomatic individuals turning threatened. $\gamma^{S}$ and $\gamma^{\mathrm{AS}}$: Rates of recovery of symptomatic and asymptomatic individuals, respectively. $\gamma^{T}$: Rate of recovery of threatened individuals (severe symptoms)

**Table 3:** Comparison of compartment inclusion across epidemiological models.

Model	Susceptible	Exposed	Asymptomatic Infectious	Symptomatic Infectious	Diagnosed	Recognised	Threatened	Recovered	Dead
SIR	✓			✓				✓	
SEIRD	✓	✓		✓				✓	✓
SEAIRD	✓	✓	✓	✓				✓	✓
SIDARTHE	✓		✓	✓	✓	✓	✓	✓	✓
Simplified SIDARTHE	✓		✓	✓			✓	✓	✓

**Table 4:** Average monthly deviations (Dev.) and proportions (Prop.) of overestimations (where underestimations are simply calculated as 1−Prop.) for each time-period in thousands.

Date	SIR Dev.	SIR Prop.	SEAIRD Dev.	SEAIRD Prop.	SIDARTHE Dev.	SIDARTHE Prop.
31 March 2020	−39.99	0	−39.91	0	−40.11	0
30 April 2020	−447.01	0	−436.90	0	−448.14	0
31 May 2020	−398.69	0	−16.68	0.3871	−406.34	0
30 June 2020	−352.35	0	1468.40	1	−396.83	0
30 April 2021	938.19	1	−87.57	0.1333	−86.70	0.0667
31 May 2021	5673.71	1	455.68	1	267.22	1
30 June 2021	13,032.57	1	899.20	1	522.53	1
31 July 2021	14,754.88	1	684.15	1	195.95	0.7586
30 November 2021	719.22	0.9667	691.04	1	−263.66	0.0667
31 December 2021	2670.63	1	−885.95	0.2258	−1562.00	0
31 January 2022	−1271.81	0.2581	−9713.99	0	−9966.65	0
28 February 2022	7009.39	1	−3496.24	0	−3559.17	0

**Table 5:** Absolute difference in the time to peak prediction (in days).

Date	SIR	SEIR	SEAIRD	SIDARTHE
1 March 2020	0	12	0	0
1 July 2020	29	0	0	22
1 November 2020	7	46	8	11
1 April 2021	81	59	15	72
1 November 2021	23	64	79	5

**Table 6:** Summary of model performance across time phases and evaluation metrics.

Model	RMSE/MAE			Estimation Orientation			Time to Peak
	Early	Mid	Late	Early	Mid	Late	Accuracy
SIR	Moderate	High	Low	Under	Over	Over-Under	High
SIRD	Moderate	High	High	Under	Over	Over	High
SEAIRD	High	Low	High	Over	Over	Under	Low
SIDARTHE	Moderate	Low	High	Under	Over-Under	Under	Low

## Data Availability

The data that supports the findings of this study is openly available in “JHU CSSE COVID-19 Dataset Repository” at https://github.com/CSSEGISandData/COVID19 (accessed on 1 September 2024).

## Appendix A. Summary of Parameter Values

In this section, we summarize the parameters, their definitions, and their associated values. We tried to unify the notation due to some similarities in the models, but there might be some differences. We note that the values of some similar parameters might be different, and that is due to the difference in the mathematical formulation, which, when calibrated to achieve R0∈[1.2,1.6], could be different.

**Table A1:** SIR.

Parameter	Definition	Value
β	Rate of transmission	0.16
γ	Rate of recovery	0.1

**Table A2:** SEIRD.

Parameter	Definition	Value
$\tilde{\beta }$	Rate of exposure to infected individuals	1
α	Probability of incubated individuals turning negative	0.1
ρ	Rate of incubated individuals turning infectious	0.16
γ	Rate of recovery	0.08
δ	Rate of mortality	0.02

**Table A3:** SEAIRD.

Parameter	Definition	Value
$\bar{\beta }$	Rate of exposure to incubated individuals	Optimized
ϕ	Probability of exposure to infection	Optimized
α	Probability of incubated individuals turning negative	Optimized
ξ	Ratio of reverting to the susceptible after recovery	Optimized
ρ	Rate of incubated individuals turning infectious	Optimized
θ	Fraction of exposed individuals becoming symptomatic	Optimized
ν	Rate of cured individuals turning positive	Optimized
γ	Rate of recovery	Optimized
δ	Rate of mortality	Optimized

**Table A4:** SIDARTHE.

Parameter	Definition	Value
$\beta^{S}$	Rate of transmission of symptomatic individuals	0.124
$\beta^{AS}$	Rate of transmission of asymptomatic individuals	0.155
ζ	Rate of asymptomatic individuals developing symptoms	0.14
μ	Fraction of symptomatic individuals turning threatened	0.017
$\gamma^{S}$	Rate of recovery of symptomatic individuals	0.1
$\gamma^{\mathrm{AS}}$	Rate of recovery of asymptomatic individuals	0.14
$\gamma^{T}$	Rate of recovery of threatened individuals (severe symptoms)	0.1
δ	Rate of mortality	0.007

## Appendix B. Additional Modeling Aspects for SIDARTHE

In the below equations, we show a modified version of the SIDARTHE model that accounts for vaccination with a rate η at which vaccinated individuals move from S to H. In addition, the parameter $\hat{r}$ corresponds to the possibility of reinfections. For COVID-19, the reinfection rate $\hat{r}=\frac{1}{120}$, which corresponds to an immunity period of about 4 months [29].

$$\begin{array}{cc} \; & \frac{dS}{dt}=\frac{-S(\beta^{S}I+\beta^{\mathrm{AS}}A)}{N}-\eta S+\hat{r}H,\\ \; & \frac{dI}{dt}=\zeta A-\left(\mu +\gamma^{S}\right)I,\\ \; & \frac{dA}{dt}=\frac{S(\beta^{S}I+\beta^{\mathrm{AS}}A)}{N}-\left(\gamma^{\mathrm{AS}}+\zeta \right)A,\\ \; & \frac{dT}{dt}=\mu I-\left(\delta +\gamma^{T}\right)T,\\ \; & \frac{dH}{dt}=\gamma^{S}I+\gamma^{\mathrm{AS}}A+\gamma^{T}T+\eta S-\hat{r}H,\\ \; & \frac{dE}{dt}=\delta T. \end{array}$$

## Appendix C. Mean Absolute Error Evaluation

We note that the results of the MAE presented in Figure A1 closely match those for the RMSE in Figure 3.
